# Silica‐coated magnetic nanoparticles labeled endothelial progenitor cells alleviate ischemic myocardial injury and improve long‐term cardiac function with magnetic field guidance in rats with myocardial infarction

**DOI:** 10.1002/jcp.28492

**Published:** 2019-04-14

**Authors:** Bo‐fang Zhang, Hong Jiang, Jing Chen, Qi Hu, Shuo Yang, Xiao‐pei Liu

**Affiliations:** ^1^ Department of Cardiology, Hubei Key Laboratory of Cardiology Renmin Hospital of Wuhan University, Cardiovascular Research Institute, Wuhan University Wuhan China

**Keywords:** endothelial progenitor cells (EPCs), magnetic field guidance, myocardial infarction (MI), silica‐coated magnetic nanoparticles

## Abstract

Low retention of endothelial progenitor cells (EPCs) in the infarct area has been suggested to be responsible for the poor clinical efficacy of EPC therapy for myocardial infarction (MI). This study aimed to evaluate whether magnetized EPCs guided through an external magnetic field could augment the aggregation of EPCs in an ischemia area, thereby enhancing therapeutic efficacy. EPCs from male rats were isolated and labeled with silica‐coated magnetic iron oxide nanoparticles to form magnetized EPCs. Then, the proliferation, migration, vascularization, and cytophenotypic markers of magnetized EPCs were analyzed. Afterward, the magnetized EPCs (1 × 10^6^) were transplanted into a female rat model of MI via the tail vein at 7 days after MI with or without the guidance of an external magnet above the infarct area. Cardiac function, myocardial fibrosis, and the apoptosis of cardiomyocytes were observed at 4 weeks after treatment. In addition, EPC retention and the angiogenesis of ischemic myocardium were evaluated. Labeling with magnetic nanoparticles exhibited minimal influence to the biological functions of EPCs. The transplantation of magnetized EPCs guided by an external magnet significantly improved the cardiac function, decreased infarction size, and reduced myocardial apoptosis in MI rats. Moreover, enhanced aggregations of magnetized EPCs in the infarcted border zone were observed in rats with external magnet‐guided transplantation, accompanied by the significantly increased density of microvessels and upregulated the expression of proangiogenic factors, when compared with non‐external‐magnet‐guided rats. The magnetic field‐guided transplantation of magnetized EPCs was associated with the enhanced aggregation of EPCs in the infarcted border zone, thereby improving the therapeutic efficacy of MI.

## 1 INTRODUCTION

Myocardial infarction (MI) remains the leading cause of morbidity and mortality worldwide, despite the progresses in treating MI from basic and clinical studies (Luscher, 2018; Shaw et al., 2018). Although, timely revascularization has been applied as the cornerstone therapy for MI, regional ischemia, and subsequent injury associated with myocardial reperfusion, could lead to heart failure, and ultimately sudden cardiac death (Bulluck, Dharmakumar, Arai, Berry, & Hausenloy, 2018; Yang et al., 2017). Therefore, exploring novel treatments to alleviate myocardial ischemia is important to improve the prognosis of patients with MI.

Cell therapy is an innovative, exciting, and promising therapeutic option for ischemic disease, which possesses the potential to fundamentally heal necrotic organs through cell regeneration (Pompilio, Nigro, Bassetti, & Capogrossi, 2015). Over the past decades, stem cell‐based therapy has been demonstrated to be effective in the treatment of many diseases, including ischemic cardiomyopathy (Burt et al., 2012; Fernandez‐Ruiz, 2017; Sasaki et al., 2006; Tang et al., 2015). Endothelial progenitor cells (EPCs) are the most commonly used cells for the cell therapy of ischemic diseases because of their mobilization, homing, and angiogenic effects (Asahara et al., 1997). Traditionally, the formation of primordial blood vessels is known as “vasculogenesis”. It is a phenomenon that has only been previously deemed to occur at the phase of embryogenesis, owing to increasing nutrition and oxygen demands with the development of embryonic organs (Jujo, Ii, & Losordo, 2008). In addition, the revascularization of ischemic or injury sites after birth has been considered to rely on the proliferation of already existing nearby mature endothelial cells, which is a process described as “angiogenesis” (Simard et al., 2017). Accumulating latest evidence suggests that EPCs differentiate into mature endothelial cells in a process similar to the mechanism of vasculogenesis after ischemic insult (Li et al., 2015). Accordingly, enhancing EPCs to home the desired region after MI to facilitate vascularization and augment blood perfusion is an attractive therapeutic strategy. The results of animal experiments were promising. However, the results of clinical trials that evaluated the efficacy of EPC‐based therapy for patients with MI are mixed (Fujita & Kawamoto, 2017). Although, the inadequate population of EPCs in peripheral blood and impaired function of EPCs in patients may be involved (Bianconi et al., 2018; Taylor, Resende, & Sampaio, 2018), the low retention of EPCs in the infarct area has been suggested to be responsible for the poor clinical efficacy of EPCs for MI (Terrovitis, Smith, & Marban, 2010). Therefore, strategies to enhance the aggregation and long‐term retention of EPCs in the ischemic myocardium may improve the therapeutic efficacy of EPC therapy for MI.

The use of nanomaterials to modify and manipulate the properties of cells has been considered as a great breakthrough in the field of nanomedicine (Overchuk & Zheng, 2018). Moreover, superparamagnetic iron oxide nanoparticle (SPION), a conventional magnetic resonance imaging contrast agent (Reddy, Arias, Nicolas, & Couvreur, 2012), has been applied to endow labeled cells magnetic property, enabling these cells to be manipulated by magnetic fields at a certain distance (Chaudeurge et al., 2012). Previous studies have indicated that magnetic forces could be used to capture magnetized endothelial cells on modified vascular grafts (Polyak et al., 2016), and SPION‐labeled mesenchymal stem cell (MSCS) and cardiosphere‐derived cells (CDCs) have been demonstrated to exert positive effects in cardiovascular diseases (Huang et al., 2013; Vandergriff et al., 2014). Furthermore, with the guidance of an external magnetic field, “magnetized EPCs” have been used to treat various ischemia‐related diseases (Chen et al., 2013; Koiwaya et al., 2011; Kyrtatos et al., 2009). On the basis of these above facts, it was hypothesized that with the guidance of an external magnetic force, the accumulation of “magnetic EPCs” in the ischemia region after MI might be significantly enhanced, thereby improving therapeutic efficacy. To confirm this hypothesis, EPCs were labeled with new nanomaterials called silica‐coated SPION, and it was explored whether this could ameliorate MI‐induced myocardial injury and improve cardiac function with the guidance of an external magnetic field.

## 2 MATERIALS AND METHODS

### 2.1 Primary EPC isolation and identification

Bone marrow mononuclear cells (MNCs) were isolated from the long bone of male syngeneic adult Sprague‐Dawley (SD) rats (weight, 150–180 g) by density gradient centrifugation using lymphocyte separation medium (MP Biomedicals, Santa Ana, CA). Then, the MNCs were separated and resuspended with EGM‐2 SingleQuots (CC‐4176; Lonza, CA), containing 2% fetal bovine serum, hydrocortisone, human fibroblast growth factor‐basic (hFGF‐B), VEGF, recombinant human‐epidermal growth factor (rhEGF), recombinant analogue insulin‐like growth factor 1 (R3‐IGF‐1), ascorbic acid, glycated albumin‐1000 (GA‐1000), and heparin. Subsequently, the cell suspension was seeded into human‐fibronectin‐coated (Sigma‐Aldrich, San Louis, MO) 60‐mm dishes and incubated at 37°C in an atmosphere containing 95% air and 5% CO_2_. After 3 days, the cell colony emerged and the non‐adherent cells in the medium were abandoned. Then, fresh EGM‐2 was added, and the cells remained incubated for another 4 days. Seven days after culture, the EPCs were characterized as spindle‐shaped and reached approximately 80% confluence. Then, these cells were trypsinized and passaged. At 3 weeks after incubation, the cultured EPCs had a typical endothelial cell‐like cobblestone appearance.

The identification of EPCs were performed by 1,1’‐dioctadecyl‐3,3,3’,3’‐tetramethylindocarbocyanine (DiI)‐labeled acetylated low density lipoprotein (Dil‐ac‐LDL; Yeasen; H7970) uptake and the fluorescein isothiocyanate (FITC)‐labeled Ulex europaeus agglutinin (UEA)‐1 (FITC‐UEA‐1; L9006; Sigma‐Aldrich) binding co‐staining experiment. Briefly, after 7 days in culture, the EPCs were washed with phosphate buffer saline (PBS) and incubated with Dil‐ac‐LDL (10 μg/ml) at 37°C for 4 hours. Then, FITC‐UEA‐1 (10 μg/ml) was added and incubated for 1 hour. Finally, the cells were incubated with 4’,6‐diamidino‐2‐phenylindole (DAPI). Differentiating EPCs were recognized as dually positive for Dil‐ac‐LDL uptake and FITC‐UEA‐1 binding when observed by fluorescence microscopy.

### 2.2 Silica‐coated magnetic iron oxide nanoparticles labeling

Silica‐coated magnetic iron oxide nanoparticles were provided by Xi'an Ruixi Biological Technology Co., Ltd (Xi'an, China). The nanoparticles were 60 nm in diameter, characterized with a 40‐nm Fe_3_O_4_ core at the center, and surrounded by a silica layer in the outer surface. Adherent EPCs were washed with PBS and incubated with nanoparticles that dissolved into serum‐free endothelial basal medium‐2 (EBM‐2) with various concentrations of 0, 10, 25, 50, 100, and 200 μg/ml for 12 hr. To identify the labeling efficiency of nanoparticles and its influence on cell vitality at different doses, Prussian blue staining and flow cytometry assay were performed, respectively. For Prussian blue staining, at the end of the co‐incubation, labeled and unlabeled EPCs on the glass coverslips were fixed with 4% formaldehyde. Then, these cells were stained with a mixture solution that consisted of 2% potassium ferrocyanide and 2% HCl solution in equal proportions for 30 min. Thereafter, cell nuclei were counterstained with nuclear fast red. The vitality of EPCs, incubated with different concentrations of nanoparticles, was measured by Annexin V‐FITC and propidium iodide double staining, as previously described (Zhang et al., 2018). The apoptosis rate was calculated as the percentage of early apoptosis plus necrosis after the ﬂow cytometric analysis. The transmission electron microscopy (TEM) study was performed to further confirm the endocytosis of the nanoparticles. The labeled EPCs were gently scraped and collected into a centrifuge tube, and fixed with electron microscope fixative fluid for 2 hr at room temperature. Then, the samples were dehydrated with graded alcohol, and the ultrathin sections were observed by TEM.

### 2.3 Impact of nanoparticles labeling on the functions of EPCs

All in vitro experiments for determining whether nanoparticle labeling would affect the normal properties of EPCs were performed at 24 hr after transfection.

#### 2.3.1 Cell proliferation and migration assays

The migration capacities of labeled and unlabeled EPCs were evaluated by the transwell chamber assay (Zhang et al., 2018). The cell number that transmigrated to the lower surface of the membrane was observed under a light microscope. The Cell Counting Kit‐8 assay was performed as previously described in two groups, to detect the proliferative ability of EPCs for 5 consecutive days (Zhang et al., 2018).

#### 2.3.2 Tube formation assay

To investigate the impact of nanoparticles labeling on the angiogenesis capacity of EPCs, a tube formation assay was performed. Then, 80 μl of Matrigel was added into the wells of a 96‐well plate. Afterward, 1 × 10^4^ labeled and unlabeled EPCs suspended into 100 μl of EBM‐2 medium were seeded into matrigel‐coated wells for 1 hr after the Matrigel solidified. At 8 hr after incubation, the tubule structure was observed under a light microscope, and the total tube length and tube lumen number in one high‐power field of each well were measured using the ImageJ software.

#### 2.3.3 Cell phenotype assay

To determine whether the incorporation with silica‐coated magnetic iron oxide nanoparticles intervened with the phenotype of EPCs, the cell surface markers of labeled and unlabeled EPCs were detected after 10 days of incubation. The EPCs in these two groups were digested and resuspended. Subsequently, these cells were incubated with antibodies of CD133 (Rabbit polyclonal CD133 Antibody; PE; NB120–16518PE), CD34 (Rat monoclonal CD34 Antibody; FITC; NB600–1071F), and VEGFR (Rabbit polyclonal VEGFR Antibody; NB100–2382AF647). Then, the percentages of these biomarkers were analyzed by fluorescence‐activated cell sorting (FACS; FACSCalibur, BD Biosciences, NJ). CD133 was regarded as a biomarker of hematopoietic stem cell lineage, whereas VEGFR and CD34 were considered as biomarkers of endothelial cell lineage.

#### 2.3.4 Cell secretion capacity assay

EPCs could synthesize and release varieties of pro‐angiogenesis cytokines, such as VEGF, IGF‐1, FDGF, SDF‐1α, and β‐FGF. The protein expression of neovascularization‐related cytokines in these two groups was measured by western blot analysis.

### 2.4 Magnetic responsibility assay of labeled EPCs in static and flowing conditions in vitro

To determine whether the external magnetic field could manipulate the magnetized EPCs, their distribution with the intervention of an external magnetic field at a static status was first explored. In brief, after 24 hr of co‐incubation, the magnetized EPCs were digested and seeded into 60‐mm dishes with or without a magnet under the bottom center of the dishes. After incubation for 24 hr, the cells were fixed with 4% formaldehyde and stained with DAPI. Cell concentration degrees at different areas were observed by fluorescence microscopy.

To better mimic the blood flow status caused by heart constriction in vitro, magnetized EPCs were resuspended with PBS and collected into 15‐ml centrifuge tubes. Then, these were located onto a swaying shaker, with or without a magnet field, and fixed on the outer surface. After 15 min, the difference between the locations of the cells from these two groups were observed.

### 2.5 Determination of the optimal number of EPCs for transplantation in vivo

Previous studies have reported that cell transplanting over a certain amount would lead to the embolization of myocardial microcirculation, and compromise the therapeutic efficacy of the cell therapy. Therefore, before the transplantation experiment, the optimal EPC amount for transplantation was determined, which was associated with the maximal cell retention in the ischemia area without leading to microvascular embolism at the first place. A concentration gradient study was designed, in which 15 non‐infarcted female rats were randomly divided into five groups, and transplanted with the following number of EPCs via the tail vein: 0, 1 × 10^5^, 5 × 10^5^, 1 × 10^6^, and 5 × 10^6^. At 24 hr after transplantation, blood samples were collected from each group, and the plasma level of cardiac troponin I (cTnI) was measured by the enzyme‐linked immunosorbent assay to determine whether any microcirculation embolism occurred, according to manufacturer's instructions.

To further verify whether the transplantation with the optimal amount of nanoparticle‐labeled EPCs has any influence on the normal physiological function of rats, serum parameters of renal function (BUN and Cr), liver function (GOT and GPT), and iron content (SI and Ferritin) were measured at 1 and 4 weeks after transplantation using commercially available assay kits.

### 2.6 Establishment of the rat MI model

Female SD wild‐type rats (SPF grade, 200–250 g) were provided by Wuhan University Experiment Animal Center. All experimental procedures were conducted in accordance with the Institutional Guidelines and Guide for the Care and Use of Laboratory Animals (NIH Publication No. 85‐23, revised 1996). The rat MI model was established, as previously described. In brief, animals were fasted for 12 hr before surgery and anesthetized with intraperitoneally injected with pentobarbital sodium (60 mg/kg). Then, a volume‐controlled small animal respirator was used to support the breathing of rats through a trachea cannula, and an electrocardiograph was used to continuously monitor for standard body electrocardiogram. Next, a left thoracotomy was performed, and a 6‐0 silk suture on a small curved needle was passed through the myocardium located approximately 2–3 mm from the junction between the left atrium and arterial cone, to ligate the left anterior descending artery (LAD). A successful MI model was confirmed by the elevated ST segment in Leads‐II and the regional paleness of the myocardial surface.

### 2.7 Experiment design in vivo

Cell therapy was performed at 1 week after MI surgery. Then, 10 sham‐operated (SO) and 50 MI female rats were divided into the following six groups: (a) SO group (*n* = 10), rats were subjected to surgical manipulation without LAD occlusion and injected with 100 μl of PBS via the tail vein; (b) MI control group (MI group, *n* = 10), rats were subjected to LAD occlusion and injected with 100 μl of PBS via the tail vein; (c) MI + EPCs transplantation group (MI + EPCs group, *n* = 10), rats were subjected to LAD occlusion and transplanted with 1 × 10^6^ EPCs suspended in 100 μl of PBS via the tail vein; (d) MI + EPCs transplantation + Magnet group (MI + EPCs + M group, *n* = 10), rats were subjected to LAD occlusion and transplanted with 1 × 10^6^ EPCs via the tail vein, followed by the application of a cylindrical magnet (0.39 T) above the infarct area of the heart for 1 hr; (e) MI + nanoparticle‐labeled EPCs transplantation group (MI + Fe‐EPCs group, *n* = 10), rats were subjected to LAD occlusion and transplanted with 1 × 10^6^ nanoparticle‐labeled EPCs via the tail vein; (f) MI + nanoparticle‐labeled EPCs transplantation + Magnet group (MI + Fe‐EPCs + M group, *n* = 10), rats were subjected to LAD occlusion and transplanted with 1 × 10^6^ nanoparticle‐labeled EPCs via the tail vein, followed by the application of a magnet above the infarct area of the heart for 1 hr.

### 2.8 Histological detection of transplanted EPC retention in myocardial tissues

To improve the reliability of the histology analysis for cell retention, in a subpopulation of rats in each group transplanted with EPCs or nanoparticle‐labeled EPCs (Fe‐EPCs), cells were also transfected with adenovirus that overexpressed enhanced green fluorescent protein (GFP) as a visible marker. At 4 weeks after cell therapy, the animals were killed, and the hearts were harvested and frozen in optimal cutting temperature (OCT) compound. Myocardial tissue samples from the border zone of the infarct area were prepared and cut into 5‐μm sections with a thickness of 100 μm. To evaluate the retention of labeled EPCs and determine whether the external magnetic field could increase their aggregation in the myocardium, Prussian blue staining was performed. In brief, the specimen sections were incubated with a mixture solution of 2% potassium ferrocyanide and 2% hydrochloric acid, and counterstained with nuclear fast red.

Previous studies have reported that nanoparticles may be stagnated in tissues because of cell death or exocytosis, causing the false‐positive phenomenon for engraftment. On this account, immunocytochemistry was performed to exhibit GFP^+^ cells in each group. Cardiomyocytes in the representative slides were marked with the primary antibody of mouse anti‐α‐actinin, whereas GFP^+^ cells were marked with the primary antibody of rabbit anti‐GFP, followed by staining with secondary antibodies, accordingly. Then, the nuclei were stained with DAPI. All images were observed using a fluorescence microscope, and the mean number of GFP^+^ cells in one high‐power field in each group was calculated.

### 2.9 Quantification of engrafted EPCs in myocardial tissues by real‐time PCR

To quantitatively analyze the retention of transplanted EPCs in the heart, real‐time polymerase chain reaction (PCR) was performed at 4 weeks after cell therapy in four cell transplantation groups. As mentioned above, EPCs isolated from male donor SD rats were transplanted to female recipients, and the cell engraftment numbers could be speculated by determining the retention of the sex determining region Y (SRY) gene located on the Y chromosome in the heart of female rats. Briefly, the whole heart was harvested, weighed, and homogenized. Then, the genomic DNA was isolated from myocardial tissues using commercial kits (DP304; TIANamp Genomic DNA Kit). The TaqMan assay (Applied Biosystems, Carlsbad, CA) was used to quantify the number of transplanted cells with the rat SRY gene as a template (forward primer: 5`‐AGCCTCATCGAAGGGTTAAAG‐3`; reverse primer: 5`‐GCTGTTTCTGCTGTAGTGGGTAT‐3`). A standard curve for the amplification multiples of rat SRY genes isolated from EPCs and cells numbers was generated. As a result, on the basis of the amplification multiples measured by PCR in each sample, the retained cell numbers could be calculated. The cell retention rate was calculated as the cell number of EPCs detected in the heart over the number of total transplanted EPCs.

### 2.10 Detection of myocardial apoptosis and fibrosis

The apoptosis of cardiomyocytes was measured by terminal deoxynucleotidyl transferase dUTP nick end labeling (TUNEL) staining, according to manufacturer's instructions (Roche Applied Science, Basle, Switzerland). Anti‐α‐actinin antibody was used to mark the cardiomyocytes, whereas red fluorescent dye and DAPI were used to label the apoptotic nuclei and entire nuclei, respectively. The apoptosis index was calculated as the ratio of TUNEL‐positive cells (red) to total cardiomyocytes (blue). Myocardial remodeling after infarction was evaluated by Masson's staining, as previously demonstrated. Areas stained in blue indicated the deposition of collagen fibrils.

### 2.11 Assessment of heart morphology and cardiac function

At 4 weeks after cell transplantation, the rat hearts collected from each group were excised and transected at the plane of the papillary muscle, followed by freezing in the OCT compound. 10 μm sections with a thickness of 100 μm were obtained, and Masson's trichrome staining was performed, according to manufacturer's instructions. Thereafter, images were acquired using a Pannoramic MIDI Slide scanner. Then, the percentage of infarcted areas in each section was calculated.

The left ventricular function at each of the indexed time points was detected by transthoracic echocardiography using the MyLab 30CV ultrasound system (Biosound Easote, Inc., Guangzhou, China). Rats were given anesthesia by inhaling isoflurane, and ultrasound images were obtained from the parasternal short axis at the level of the mid‐papillary muscle from at least three separate cardiac cycles. Then, left ventricular ejection fraction (LVEF) and left ventricular fractional shortening (LVFS) were measured.

### 2.12 Determination of capillary density and neovascularization capacity of EPCs at the microcirculation level

To investigate the capillary density of different groups, the endothelial cells of representative sections were stained with the antibody of CD31. Moreover, because previous studies have indicated that transplanted EPCs could differentiate into mature endothelial cells and participate into the process of neovascularization, the antibody of GFP was used to mark endothelial cells that differentiated from the transplanted EPCs. After incubation with the secondary antibodies, images were photographed under a fluorescence microscope. The number of both CD31 and GFP‐positive cells were counted in at least three randomly high‐power fields.

### 2.13 Quantitative RT‐PCR and western blot analyses

Quantitative reverse transcription polymerase chain reaction (qRT‐PCR) was performed, as previously described in studies. Glyceraldehyde 3‐phosphate dehydrogenase (GAPDH) served as an internal control. The primer sequences for the real‐time RT‐PCR are listed, as follows: Collagen I forward: 5′‐TGACTGGAAGAGCGGAGAGT‐3′, Collagen I reverse: 5′‐GAATCCATCGGTCATGCTCT‐3′; Collagen III forward: 5′‐TTTGTGCAATGTGGGACCTG‐3′, Collagen III reverse: 5′‐AATGGGATCTCTGGGTTGGG‐3′; TGF‐β forward: 5′‐CACTCCCGTGGCTTCTAGTG‐3′, TGF‐β reverse: 5′‐GGACTGGCGAGCCTTAGTTT‐3′; GAPDH forward: 5′‐ACAGCAACAGGGTGGTGGAC‐3′, GAPDH reverse: 5′‐TTTGAGGGTGCAGCGAACTT‐3′.

For the western blot assay, the cells or myocardial samples were lysed, and proteins were extracted according to the specification of manufacturers. Total proteins were separated by 10% sodium dodecyl sulfate polyacrylamide gel electrophoresis, and electrophoretically transferred onto polyvinylidene ﬂuoride membranes (Millipore, Billerica, MA). Then, 5% nonfat dried milk were used to block the nonspecific binding sites in the membranes, and the membranes were incubated with the primary antibodies of VEGF (BS6496; Bioworld, MN), IGF‐1 (BA0939; Boster, Wuhan, China), FDGF (Ab16829; Abcam, Cambridge, British), SDF‐1α (E‐AB‐32858; Elabscience, Wuhan, China), β‐FGF (bs‐0217R; Bioss, Beijing, China), ANP (DF6479; Affinity), BNP (Ab19465; Abcam), Bax (Ab32503; Abcam), and Bcl‐2 (Ab32124; Abcam) overnight, followed by incubation with the corresponding secondary antibodies. The protein bands were visualized using an enhanced chemiluminescence system (Thermo Fisher Scientific, Inc., MA). GAPDH was used as an internal loading control.

### 2.14 Statistical analysis

Continuous values were presented as mean ± standard deviation. The Student's *t* test was used for comparisons between two groups. One‐way analysis of variance was used for comparisons among multiple groups, and Student–Newman–Keuls (SNK)‐q tests were used for subsequent analyses between multiple comparisons. *p* value of <0.05 was considered statistically significant. All statistical analyses were performed using SPSS 19.0 software.

## 3 RESULTS

### 3.1 EPC identification

**Figure 1:**
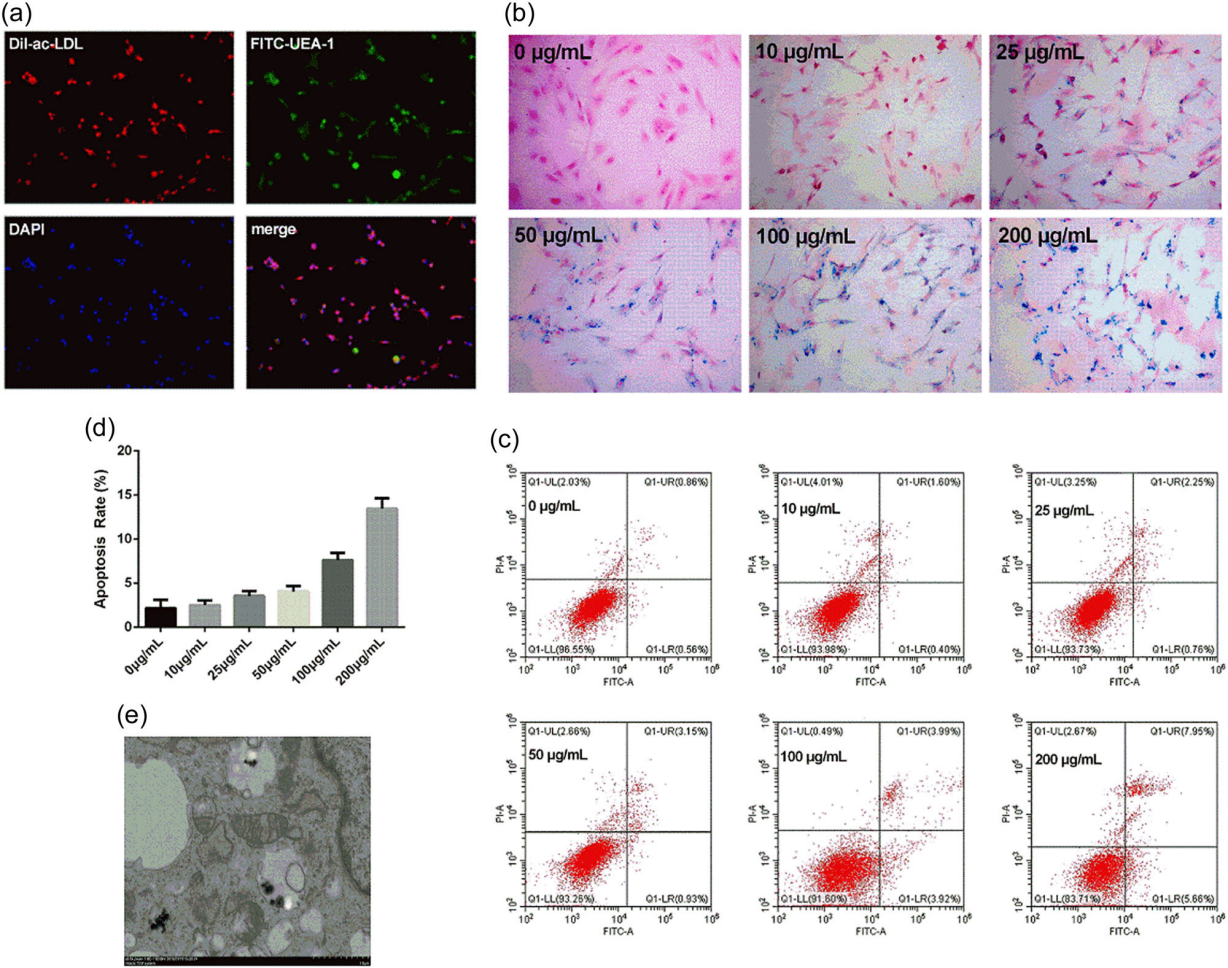
(a) The representative images for the identification of endothelial progenitor cells (EPCs). The differentiating EPCs were positive for both Dil‐ac‐LDL uptake (red) and FITC‐UEA‐1 binding (green). (b) Representative EPCs for Prussian blue staining at different nanoparticle concentrations. (c,d) The apoptosis rate of EPCs when incubated with various doses of nanoparticles. (e) Transmission electron microscopy (TEM) images of nanoparticle‐labeled EPCs at a concentration of 50 μg/ml. Dil‐ac‐LDL: 1,1’‐dioctadecyl‐3,3,3’,3’‐tetramethylindocarbocyanine (DiI)‐labeled acetylated low density lipoprotein; FITC‐UEA‐1: fluorescein isothiocyanate‐labeled ulex europaeus agglutinin [Color figure can be viewed at wileyonlinelibrary.com]

As shown in Figure 1a, at the 7th day of incubation, almost all adherent spindle‐like cells in the field of view were positive for both Dil‐ac‐LDL uptake (red) and FITC‐UEA‐1 binding (green). These results confirm that the isolated cells were exactly differentiating EPCs.

### 3.2 Labeling of EPCs with magnetic iron oxide nanoparticles

The incorporation of nanoparticles and EPCs were achieved by endocytosis. To confirm the optimal labeling condition, labeling efficiency was determined by Prussian blue staining, and EPC vitality was further measured at various nanoparticle concentrations by the ﬂow cytometric analysis. As shown in Figure 1b, the blue stained particles in the cytoplasm of labeled EPCs could be observed. In addition, the endocytosis of nanoparticles was presented in a dose‐dependent manner. However, as shown in Figures 1c,d, along with the increasing concentrations of nanoparticles, the apoptosis rates of EPCs increased, accordingly. It was found that cells incubated with nanoparticles at 50 μg/ml for 12 hr exhibited a relatively higher uptake rate, but with lower cytotoxicity. Accordingly, 50 μg/ml was chosen as the optimal concentration used in the subsequent experiment. The distribution of nanoparticles in EPCs at this concentration was observed by TEM, which revealed that these nanoparticles were located in the endosomal vesicles in the cytoplasm (Figure 1e).

### 3.3 Nanoparticles labeling exerts minimum adverse effects on normal functions of EPCs

**Figure 2:**
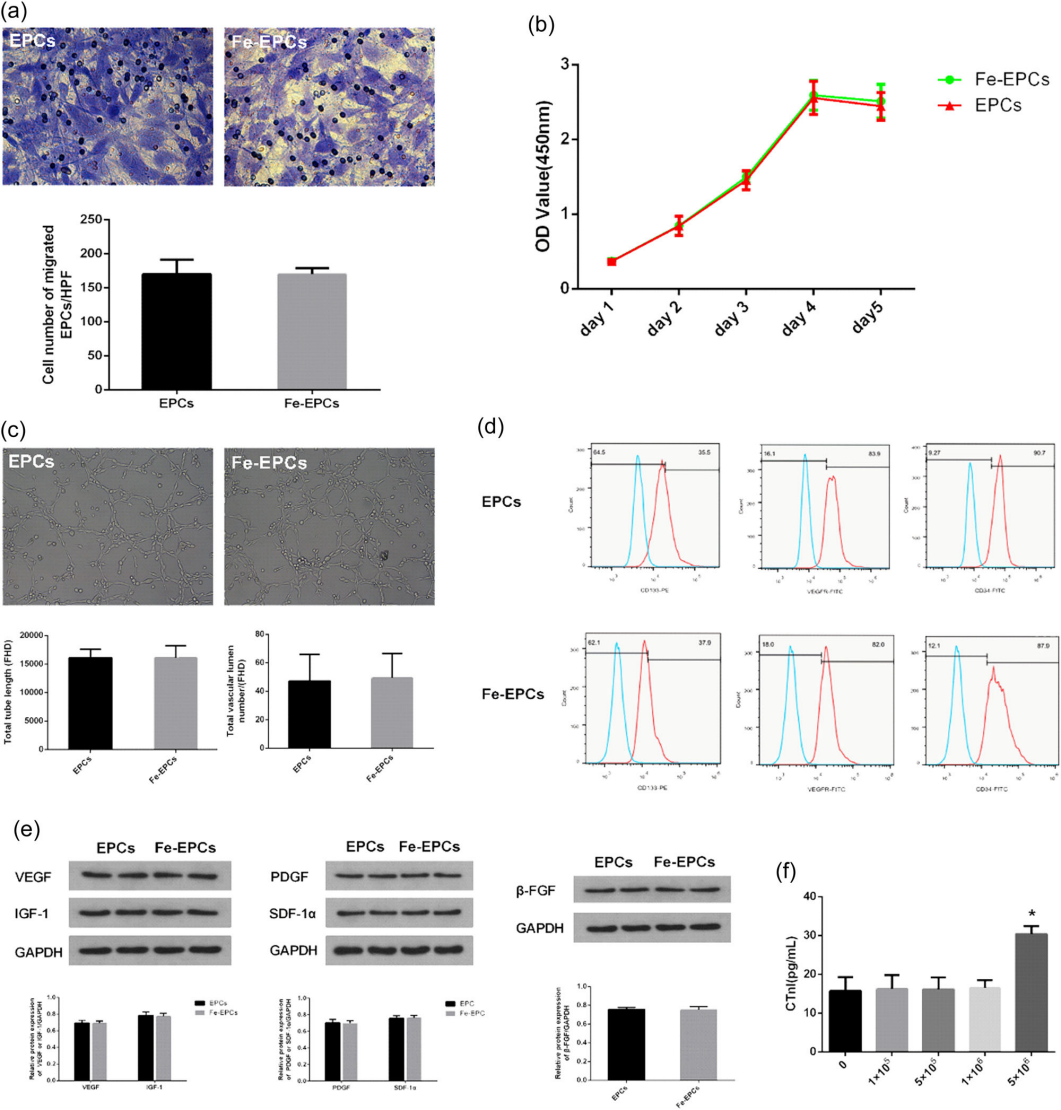
(a) Representative images and bar graphs of the transmigrated cell number in the labeled and unlabeled endothelial progenitor cell (EPC) groups (×200). (b) Proliferation curve of labeled and unlabeled EPCs in the successive five days. (c) Representative images of tube formation capacities in the labeled and unlabeled EPC groups (×200). The bar graphs show that there was no statistical difference in total tube length and total vascular lumen number between the two groups (*p* > 0.05). (d) Representative histograms of the cell phenotype of labeled and unlabeled EPCs in the FACS analysis. (e) Western blot analysis of the expression of VEGF, IGF‐1, FDGF, SDF‐1α, and β‐FGF in the labeled and unlabeled EPC groups. (f) Plasma levels of cTnI when transplanted with different doses of EPCs, and values were expressed as mean ± standard deviation (*SD*). *p < 0.05 versus the other group. cTnI: cardiac troponin I; FACS: fluorescence‐activated cell sorting [Color figure can be viewed at wileyonlinelibrary.com]

As shown in Figure 2a, at 12 hr after incubation, the transmigrated cell number in the Fe‐EPCs group was similar with the number in the EPCs group (*p* > 0.05). Similarly, the proliferation curve of the cells in the labeled and unlabeled groups were not statistically different in the successive 5 days (Figure 2b; *p* > 0.05). These results indicated that labeling with nanoparticles has no significant impact on cell proliferation and migration ability.

Neovascularization is of an important capacity of endothelial cell lineage. To determine whether nanoparticle labeling compromised the angiogenesis ability of EPCs in vitro, a tube formation assay was performed. As shown in Figure 2c, no significant differences in total tube length or total vascular lumen number were detected between cells from the two groups (*p* > 0.05). In addition, the cell phenotype of labeled and unlabeled cells was determined. As expected, the expression of biomarkers, such as CD133, CD34, and VEGFR, in cells from these two groups were practically unanimous (Figure 2d; *p* > 0.05). Cell synthesis and secretion capacity were also explored by analyzing the protein expression of angiogenic and cardiac protective factors. As shown in Figure 2e, no significant differences were observed regarding the protein levels of VEGF, IGF‐1, FDGF, SDF‐1α, or β‐FGF between labeled and unlabeled cells (*p* > 0.05).

### 3.4 External magnetic fields could manipulate the distribution and affect the aggregation of labeled EPCs in vitro

**Figure 3:**
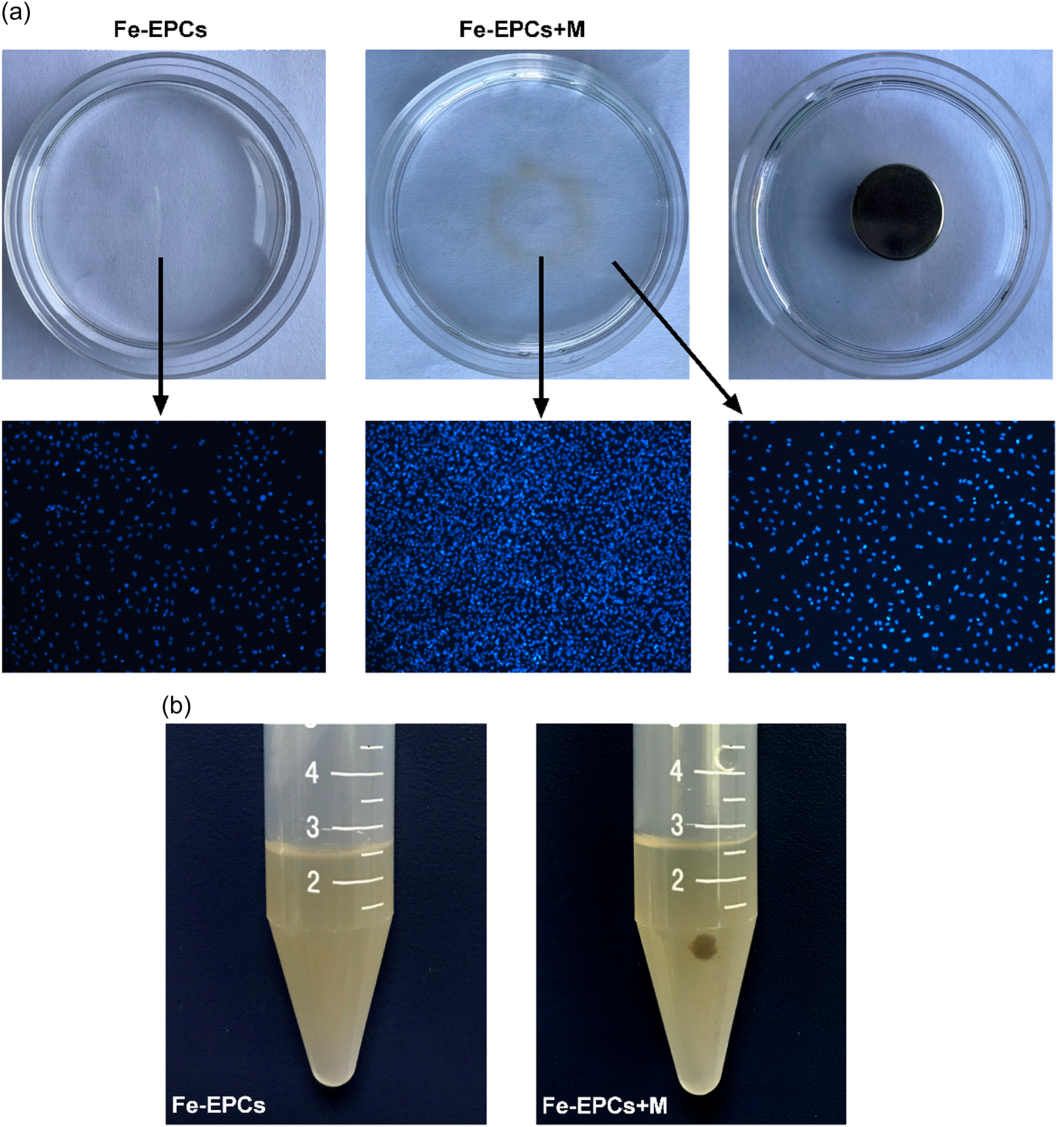
(a) These representative images show the distribution of nanoparticle‐labeled EPCs with or without an external magnetic field at static condition. (b) These representative images show the distribution of nanoparticle‐labeled EPCs with or without an external magnetic field at flowing condition. EPC: endothelial progenitor cell [Color figure can be viewed at wileyonlinelibrary.com]

To investigate the magnetic responsibility of nanoparticle‐labeled EPCs, their distributions with or without the guidance of an external magnetic field was observed. As shown in Figure 3a, a distinct brown circle could be observed in the middle of the dish with a magnet beneath its lower surface. Indeed, much more nucleus were observed in the magnet located area, when compared with the group without a magnet (*p* < 0.05). Moreover, the magnetized EPCs also exhibited satisfying magnetic responsibility in turbulent status. As shown in Figure 3b, a distinct small cell condensate was formed when a magnet was applied on the outer surface of the tube. However, the cell resuspension remained homogeneous in the group without the application of a magnet. These results indicate that the labeled EPCs could be manipulated by an external magnetic field.

### 3.5 Number of cells for transplantation

To determine the optimal number of EPCs for transplantation in vivo, serum cTnI levels were measured at different cell transplantation concentrations. As shown in Figure 2f, serum cTnI level dramatically increased when transplanted with 5 × 10^6^ EPCs, indicating that transplanting this amount of EPCs could obstruct the myocardial microcirculation. Accordingly, 1 × 10^6^ was considered as the “safe amount” of EPCs for transplantation. To further evaluate whether this amount of EPCs for transplantation would affect the normal physiological functions of rats, the serum markers of renal function and liver function, and the metabolic state of iron element were measured, accordingly. As shown in Table 1, these indicators were not significantly affected after 1 and 4 weeks of cell transplantation. Consequently, 1 × 10^6^ was chosen as the optimum number of cells for the subsequent in vivo experiment.

**Table 1:** Serum markers of renal function and liver function, and the metabolic state of the iron element after 1 and 4 weeks of cell transplantation

		After 7 days			After 28 days		
		PBS(*n* = 3)	Fe‐EPCs(*n* = 3)	*P*	PBS(*n* = 3)	Fe‐EPCs(*n* = 3)	*P*
LDH	(U/l)	455.3 ± 1.7	463.2 ± 8.8	NS	458.5 ± 13.2	482.2 ± 12.2	NS
BUN	(mmol/l)	18.91 ± 2.64	21.54 ± 2.20	NS	28.86 ± 3.59	29.77 ± 2.94	NS
Cr	(μmol/l)	30.90 ± 2.48	32.64 ± 2.07	NS	41.94 ± 3.39	43.76 ± 1.66	NS
GOT	(mg/l)	91.42 ± 0.69	95.60 ± 2.83	NS	98.69 ± 0.72	103.0 ± 4.06	NS
GPT	(U/l)	33.33 ± 0.44	34.80 ± 1.05	NS	39.40 ± 0.46	41.34 ± 0.77	NS
SI	(mg/l)	16.76 ± 0.24	17.73 ± 0.24	NS	18.83 ± 0.33	20.50 ± 0.89	NS
Ferritin	(ng/ml)	75.28 ± 0.56	71.84 ± 0.74	NS	76.83 ± 0.68	78.68 ± 0.56	NS

*Note*. EPCs: endothelial progenitor cells; PBS: phosphate buffered saline.

### 3.6 External magnetic fields dramatically increase the long‐term retention of labeled EPCs in the infarcted border zone

**Figure 4:**
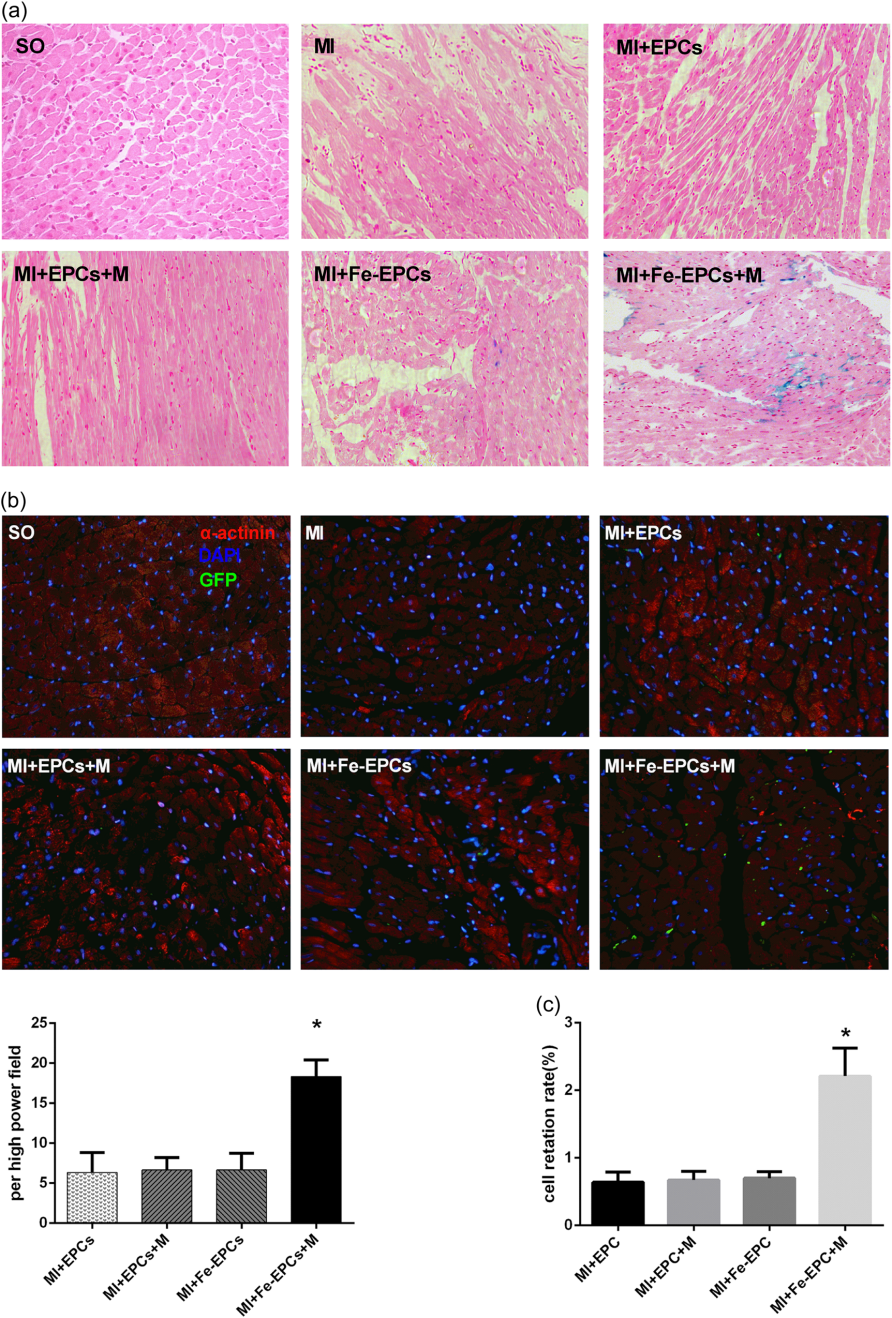
(a) Representative images of the Prussian blue staining of myocardial tissues in the different groups (×200). (b) Representative images of myocardial tissue and GFP labeled EPC double staining (×400), and the bar graph of the cell number of GFP^+^EPCs in the different groups. The α‐actinin labeled cardiomyocytes (red); DAPI‐labeled nuclei of cardiomyocytes (blue); GFP labeled EPCs (green). The values were expressed as mean ± standard deviation (*SD*). *p < 0.05 versus the other group. (c) The cell retention rate of these different groups was analyzed by RT‐PCR. The values were expressed as mean ± *SD*. *p < 0.05 versus the other group. DAPI: 4’,6‐diamidino‐2‐phenylindole; EPCs: endothelial progenitor cells; GFP: green fluorescent protein; qRT‐PCR: quantitative reverse transcription polymerase chain reaction; SO: sham‐operated [Color figure can be viewed at wileyonlinelibrary.com]

As shown by the results of the histological examination, magnetized EPCs that accumulated in the myocardium were positive for Prussian blue staining. However, the unlabeled EPCs could not be stained by Prussian blue. Figure 4a indicates that the Prussian blue^+^ area in the group with an external magnetic field was much larger than that without an external magnetic field (*p* < 0.05). To eliminate the error of possible “false‐positive phenomenon”, cells that expressed GFP by adenovirus transfection were used to trace the retention of transplanted EPCs. As shown in Figure 4b, at 4 weeks after cell therapy, the MI + Fe‐EPCs + M group had the largest number of GFP^+^ cells. Interestingly, the GFP^+^ cell number in one high‐power field was not statistically different among the MI + EPCs group, MI + EPCs + M group, and MI + Fe‐EPCs group (*p* > 0.05).

These results above were highly consistent with the data of the RT‐PCR. As shown in Figure 4c, the retention rate in the MI + Fe‐EPCs + M group was approximately 2.2 ± 0.24% at 4 weeks after cell therapy, which was much higher than that in the MI + Fe‐EPCs group (0.70 ± 0.06%) at the same time point (*p* < 0.05). Moreover, these retention rates were not statistically different among the MI + EPCs group, MI + EPCs + M group and MI + Fe‐EPCs group (*p* > 0.05), but were consistent in terms of the number of GFP^+^ cells in these three groups. These results demonstrate that magnetic field guidance could significantly increase the retention of magnetized EPCs in the ischemic myocardium. These above results also indicate that magnetic iron oxide nanoparticles labeling had no obvious influence on the engraftment of EPCs, and that the external magnetic field has no significant impact on the retention of unlabeled EPCs.

### 3.7 Magnetic guiding significantly attenuated myocardial apoptosis and fibrosis

**Figure 5:**
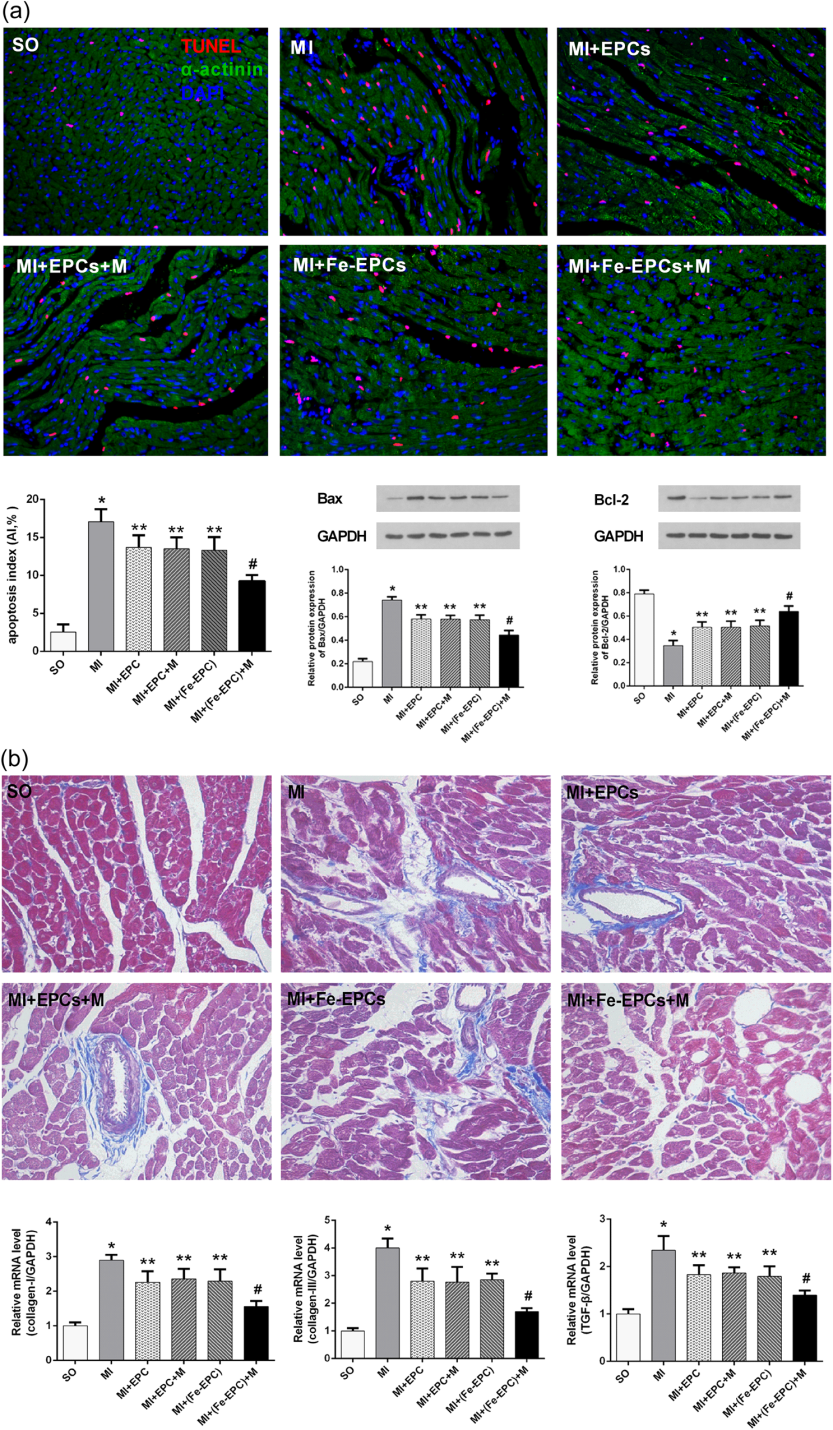
(a) Representative images of TUNEL staining in the different groups (×200): DAPI‐labeled nuclei of cardiomyocytes (blue); α‐actinin labeled cardiomyocytes (green); TUNEL‐labeled nuclei of apoptotic cardiomyocytes (red). The Western blot analysis of the expression of Bax and Bcl‐2 in the different groups is shown. (b) Representative images of the Masson staining (×200), and the mRNA expression of Collagen I, Collagen III, and TGF‐β in the different groups. The values were expressed as mean ± standard deviation (*SD*). *p < 0.05 versus the SO group; **p < 0.05 versus the MI group; *^#^p* < 0.05 versus the MI + Fe‐EPCs group. DAPI: 4’,6‐diamidino‐2‐phenylindole; EPCs: endothelial progenitor cells; GAPDH: glyceraldehyde 3‐phosphate dehydrogenase MI: myocardial infarction; mRNA: messenger RNA; SO: sham‐operated; TUNEL: terminal deoxynucleotidyl transferase dUTP nick end labeling [Color figure can be viewed at wileyonlinelibrary.com]

As shown in Figure 5a, although MI led to severe apoptosis, significantly less TUNEL^+^ cells were observed in all EPC transplantation groups, and the number of TUNEL^+^ cells in the MI + Fe‐EPCs + M group was further reduced, when compared with the MI + Fe‐EPCs group (*p* < 0.05). In addition, magnetic guidance could further decrease the proapoptotic protein level of Bax, but increase the antiapoptotic protein level of Bcl‐2. The Masson's staining result revealed that magnetic guidance could further reduce the fibrosis area (Figure 5b). Similarly, the messenger RNA (mRNA) levels of fibrosis markers, such as Collagen I, Collagen III, and TGF‐β, were significantly decreased in all cell transplantation groups. More important, these factors were of the lowest expression in the myocardium in the MI + Fe‐EPCs + M group.

### 3.8 Magnetic targeting significantly reduces infarcted size and improves cardiac function after MI

**Figure 6:**
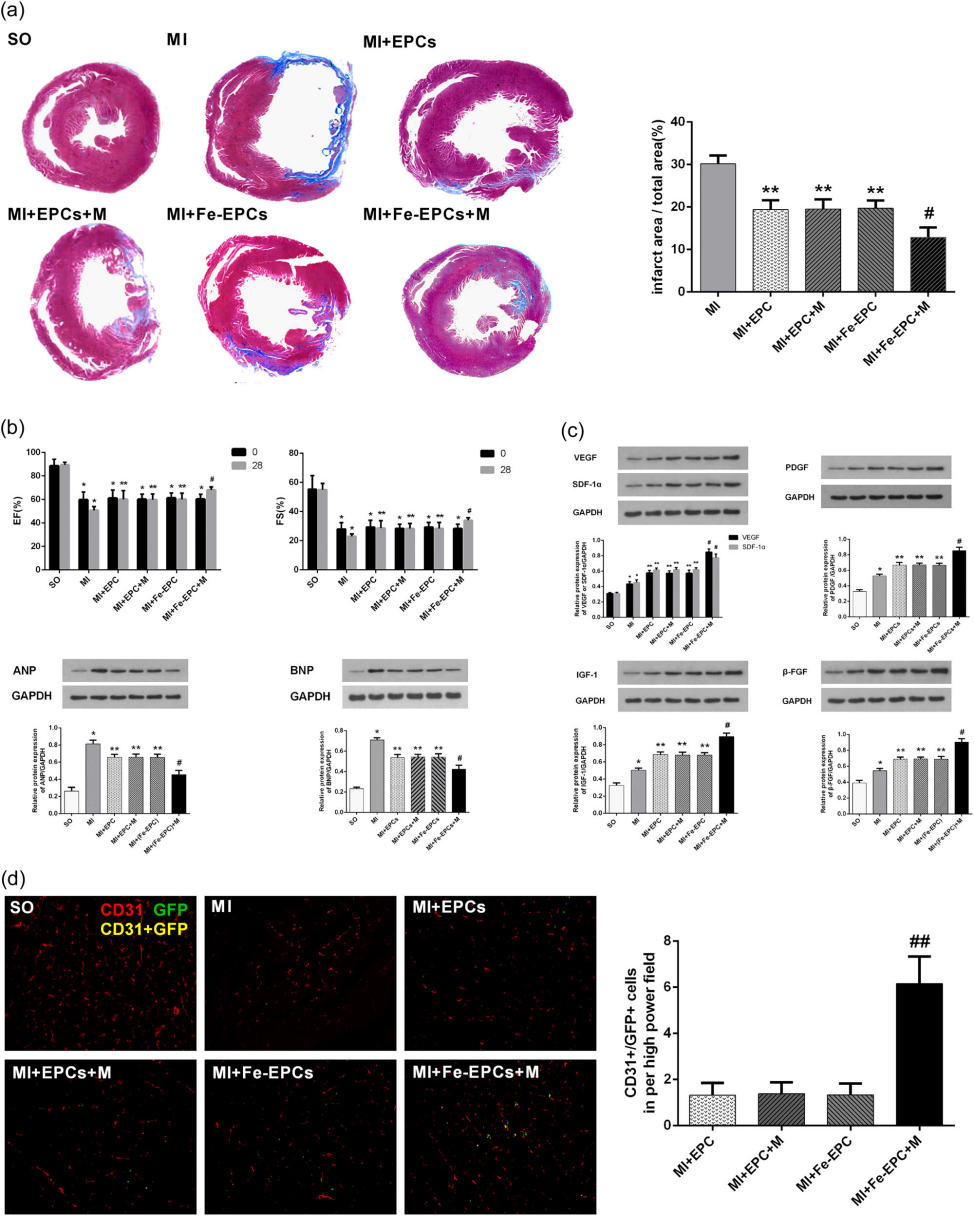
(a) Representative Masson staining images of the heart papillary muscle cross‐sectional scan images, and infarct rate of the different groups. (b) Upper panel: Bar graphs of LVEF and LVFS before and at 4 weeks after the transplantation of EPCs in the different groups. Lower panel: Western blots analysis of the expression of ANP and BNP in the different groups. (c) Western blot analysis of the expression of VEGF, IGF‐1, FDGF, SDF‐1α, and β‐FGF in the different groups. (d) Representative images of CD31 and GFP labeled EPC double staining (×200), and the bar graph of the cell number of GFP^+^ and CD31^+^ in the different groups. CD31 labeled capillary density (red); GFP labeled EPCs (green). The values were expressed as mean ± standard deviation (*SD*). *p < 0.05 versus the SO group; **p < 0.05 versus the MI group; ^*#*^ *p* < 0.05 versus the MI + Fe‐EPC group; ^##^ *p* < 0.05 versus the other group. EPC: endothelial progenitor cell; GFP: green fluorescent protein; LVEF: left ventricular ejection fraction; LVFS: left ventricular fractional shortening; MI: myocardial infarction; SO: sham‐operated [Color figure can be viewed at wileyonlinelibrary.com]

To determine whether increased EPC retention could further ameliorate myocardial remodeling and improve cardiac function, heart morphology and echocardiography were measured. At 4 weeks after cell therapy, rats in the MI group exhibited the most serious ventricular dilatation, the most extensive infarct area, and the thinnest left ventricular wall thickness. Conversely, all groups that accepted cell therapy had ameliorated myocardial remodeling and preferable cardiac morphology. Interestingly, rats in the MI + Fe‐EPCs + M group exhibited the best protective efficacy, with a minimal infarct size and the most compact ventricular structure (Figure 6a). At 1 week after LAD occlusion, LVEF and LVFS in all surgical groups were similar, indicating no difference in the cardiac function after the operation. At 4 weeks after cell therapy, the cardiac function of the PBS treatment group was dramatically exacerbated, whereas the levels of LVEF and LVFS in the MI + EPCs group, MI + EPCs group, and MI + Fe‐EPCs group were approximately maintained at baseline levels. However, cardiac function in the MI + Fe‐EPCs + M group further improved, exhibiting a prominent therapeutic effect (Figure 6b). Meanwhile, no significant difference in cardiac morphology and function was observed among rats in the MI + EPCs group, MI + EPCs + M group and MI + Fe‐EPCs group (*p* > 0.05), which again demonstrates that magnetic iron oxide nanoparticles labeling has no significant adverse impact on the normal function of EPCs.

### 3.9 Magnetic guidance could facilitate neovascularization and increase capillary density in the infarct border zone

The cells, that were both CD31 and GFP‐positive, confirmed that endothelial cells were differentiated from the transplanted EPCs. As shown in Figure 6d, among all EPC transplanted groups, cells from the MI + Fe‐EPCs + M group had the highest capillary density. In addition, it also had the largest CD31^+^/GFP^+^ cell number, indicating that more endothelial cells were differentiated from the transplanted EPCs in this group. However, CD31^+^/GFP^+^ cells in the other three cell therapy groups were rarely observed. Furthermore, the expression of pro‐angiogenesis proteins VEGF, IGF‐1, FDGF, SDF‐1α, and β‐FGF were also observed. As shown in Figure 6c, EPC transplantation could increase the expression of these pro‐angiogenesis cytokines in the infarcted border zone. More importantly, magnetic guidance could further upregulate these cytokine levels. However, no statistic difference in CD31^+^/GFP^+^ cell and pro‐angiogenesis proteins expression were found among the MI + EPCs group, MI + EPCs + M group, and MI + Fe‐EPCs group (*p* > 0.05), which demonstrate that magnetic iron oxide nanoparticles labeling exerted no significant adverse influence on the differentiation and secretion functions of EPCs.

## 4 DISCUSSION

The pivotal issue for the survival of ischemia organs rest with the formation of new blood vessels, which is especially true when it comes to MI (Losordo & Dimmeler, 2004). As a physical approach, magnetic force has been reported to aggregate magnetized therapeutic agents, such as drugs or cells, longstanding enough within a designated area (Koiwaya et al., 2011; Polyak et al., 2016; Watermann & Brieger, 2017). To overcome the low EPC retention at the ischemic myocardium because of the heart beat and rapid blood flow scouring (Fernandez‐Ruiz, 2017; Terrovitis, Smith, & Marban, 2010), in the current study, EPCs were labeled with silica‐coated magnetic iron oxide nanoparticles, the effective integration confirmed, and the potential therapeutic efficacy of magnetized EPCs with the external magnetic guidance was explored. Furthermore, it was found that nanoparticles at a concentration of 50 μg/ml exerted no obvious adverse impact on the proliferation, migration, tube formation, phenotype, and secretion capacity of EPCs. Moreover, the subsequent in vivo experiment demonstrated that the number of labeled EPCs significantly increased in the infarcted border zone with the guidance of an external magnetic field. More importantly, the increased cell retention of EPCs contributed to the attenuation of myocardial apoptosis and remodeling, with improved cardiac function and reduced infarct size. To the best of our knowledge, this report is the first to elaborate that an external magnetic field could remarkably accelerate the retention of magnetized EPCs in the ischemic myocardium, which in turn could alleviate myocardial injury and improve long‐term cardiac function.

An important advantage of the present experimental study was the application of silica‐coated magnetic iron oxide nanoparticles, instead of traditional SPION, for the labeling of EPCs. The material possesses multiple advantages. Silica is classified as “Generally Recognized as Safe” by the FDA (Chen et al., 2018). With a silica layer wrapped around the Fe_3_O_4_ core with a diameter of 60 nm, the nanoparticles exhibited excellent biocompatibility and higher cell endocytosis efficiency without any obvious side effects. In addition, compared with SPION, the silica outer surface endowed the rigid structure of nanoparticles, thereby keeping it insensitive and stable, regardless of the changes in pH and temperature. Moreover, the silica layer acts as a protective shell, which prevents the Fe_3_O_4_ core from quick degradation. Finally, the particular structure of the material not only maintains the long‐term reactiveness of the particle to the external magnetic field, but also prevents the release of a large amount of iron element, which may result in cell cytotoxicity (Li et al., 2013; Watermann & Brieger, 2017). In the current study, silica‐coated magnetic iron oxide nanoparticle‐labeled EPCs exhibited excellent magnetic responsibility and biocompatibility. Therefore, the investigators consider that this material will be safer and more effective for clinical applications.

Stem cell‐based cell therapy has become an attractive and potential treatment strategy for MI (Hou, Kim, Woo, & Huang, 2016; Ledney et al., 1985; Tompkins, Natsumeda, Balkan, & Hare, 2017). EPC is one kind of mononuclear cell that mainly exists in the bone marrow and peripheral blood with high proliferation capability, which express both hematopoietic stem cell and endothelial cell markers (Goligorsky, 2014; Medina et al., 2017; Menasche, 2018). The number of circulating EPCs has been considered as a biomarker of cardiovascular disease (Bianconi et al., 2018). Furthermore, it has been confirmed that EPCs could not only be mobilized and homed to the injury site and differentiate into mature endothelial cells, but also secrete protective cytokines to facilitate surrounding endothelial cells to extend towards the injured sites and renovate the integrity of endothelial cell layer, playing a key role as a self‐repair mechanism in MI (Goligorsky, 2014; Jujo, Ii, & Losordo, 2008). Moreover, EPCs could differentiate into vascular smooth muscle cells or even cardiomyocytes under certain conditions (Kawamoto et al., 2006; Lopez‐Ruiz et al., 2014). All of the above pathophysiological mechanisms make EPC‐based cell therapy a potentially effective treatment for MI. Although this has been proven to be promising in theory and in preclinical studies, the therapeutic efficacy of EPC‐based cell therapy for MI in clinical trials retrieved inconsistent results. The inadequacy of accumulation of EPCs in the ischemic myocardium has been recognized as a major cause of treatment failure in EPC‐based cell therapy for MI. Therefore, enhancing the retention of EPCs in the ischemic myocardium is particularly important for improving the therapeutic efficacy of EPC‐based cell therapy for MI.

The dose and curative effect are often concentration dependent in pharmaceutical studies. However, this is not the situation for cell therapy. In fact, researchers have demonstrated that the transplantation of excessive cells would obstruct the microcirculation. Moreover, the external magnetic field would further increase the aggregation of magnetized cells, thereby exacerbating the risk of coronary embolism (Huang et al., 2013; Tompkins et al., 2017). In the current study, 1 × 10^6^ EPCs were transplanted, which displayed no obvious adverse influence to the normal physiological function of rats. The time point of EPC infusion after MI was another critical issue. Indeed, there is a paradox in cell transfer time selection, and myocardial interstitial edema and severe inflammatory response persists at the acute phase after MI. Therefore, cell transplantation efficiency at this time window might be restricted. However, EPCs exhibited a limited homing efficiency at 7–9 days after MI (Mu et al., 2016; Terrovitis, Smith, & Marban et al., 2010). Therefore, 1 week after MI was identified as the ideal time point to implant EPCs. In addition, the optimal EPC delivery route remains uncertain. Furthermore, the intracoronary artery route could directly infuse infarct‐related coronary arteries with EPCs. However, non‐perfused areas would not be benefited. Intramyocardial injection has been associated with the highest cell retention rate, but the invasive nature of this strategy would increase the risk of mortality, and has the potential to induce arrhythmia. Although merely a small amount of injected cells could home to the site of interest via intravenous routes, it is the least invasive approach and practical in daily clinical practice (Fakoya, 2017). As a translational medical study, intravenous routes were used to transplant EPCs in the current study. Previous studies have indicated that 10 min was long enough for the external magnetic field to act on the magnetized cells and accumulate these at the injury site (Cheng et al., 2012; Vandergriff et al., 2014). To maximize the attraction capability of the magnetic force, the magnet was placed above the ischemia region until the rats woke up from anesthesia, and this process lasted for approximately 1 hour.

Early studies have indicated that the therapeutic effect of EPCs mainly relay on its direct transdifferentiation into endothelial cells to keep the integrity of the endothelial layer, which is called, the “direct mechanism”, whereas subsequent studies have suggested that pro‐angiogenesis cytokines released by engrafted EPCs at the injury site to facilitate the self‐repair and extension of endothelial cells might play a more important role, namely, the “indirect mechanism”. In the current study, it was found that the transplantation of EPCs could ameliorate MI‐induced injury through both of the above mechanisms. The existence of GFP^+^/CD31^+^ cells demonstrates that transplanted EPCs could directly differentiate into endothelial cells. However, the number of GFP^+^/CD31^+^ cells is rare, indicating that the direct mechanism may be less important. Furthermore, it was found that pro‐angiogenesis cytokines, such as VEGF, IGF‐1, FDGF, SDF‐1α, and β‐FGF, were extremely abundant at the ischemia region. As a result, it was speculated that this indirect mechanism might contribute more therapeutic benefits.

There were several limitations in the current study. First, the EPCs transplanted in the experiment were early outgrowth EPCs. Different results might be obtained if late outgrowth EPCs would be used. Second, cell transplantation was performed only after MI. Some clinical studies have revealed that cell therapy could improve the cardiac function in the short term. However, no significant improvement in cardiac function could be observed in the long run, such as at 6 months after implantation. As the retention of stem cells in the body tends to decrease over time, repeated transplantation might enhance its long‐term efficacies. Third, mere changes in the cardiac function at 4 weeks after EPC implantation were observed. Fourth, for patients with cardiovascular diseases, the normal function of EPCs would be compromised. As a result, the strategy of EPC auto‐transplantation may probably be not as effective as that in the animal experiment. Fifth, a rat model of MI was used in the current study. Large animal experiments are still needed to determinate the corresponding EPC transplantation number and magnetic field parameter before clinical application. In addition, it should be noted that multiple mechanisms mediate cell loss after transplantation. With this rationale, a combination of magnetic targeting and multiple joint mechanisms, such as gene modification and drugs, to enhance cell engraftment capability, might be more effective.

In conclusion, the current study demonstrates that the magnetic field‐guided transplantation of magnetized EPCs is associated with the enhanced aggregation of EPCs in the infarcted border zone, thereby improving their therapeutic efficacy for MI. Moreover, the safety of silica‐coated magnetic iron oxide nanoparticle‐labeled EPCs was confirmed in the current study. These results suggest that the magnetic field‐guided transplantation of magnetized EPCs may become a feasible new therapeutic strategy for MI.

## AUTHOR CONTRIBUTIONS

B‐F.Z., J.C., and H.J. have made substantial contributions to conception and design; B‐F.Z. and J.C. have done acquisition of data; Q.H. and S.Y. have done the analysis and interpretation of the data; B‐F.Z. and X‐P.L. were involved in drafting the manuscript; H.J. and J.C. revised it critically for important intellectual content.

## CONFLICT OF INTERESTS

The authors declare that there are no conflict of interests.
